# Supplementary material for: Explore or reset? Pupil diameter transiently increases in self-chosen switches between cognitive labor and leisure in either direction
Source: Cogn Affect Behav Neurosci. 2019 Jun 17;19(5):1113–28. doi: 10.3758/s13415-019-00727-x (PMC6785586; doi:10.3758/s13415-019-00727-x)
Supplement: Supplementary file 1 — (DOCX 213 kb) [file 13415_2019_727_MOESM1_ESM.docx]

Supplementary Online Materials

Explore or reset? Pupil diameter transiently increases in self-chosen switches between cognitive labor and leisure in either direction

Appendix S1

Participant- and Trial-Based Data Inclusion in Study 1

Four of the 35 participants were excluded from all analyses in accordance with our pre-registered criteria: Two of them did never switch to the leisure task, but only performed the labor task; one performed below 50% accuracy in the labor task, and one participant’s eye-tracking data were unusable due to a high percentage of missing values.

For the *pre-registered* analyses of pupil data, we selected the last ten but one trials before each labor-to-leisure switch. The adjacent trials preceding the same switch were treated as belonging to the same *bout*, with trials nested in bouts and bouts nested in participants, yielding 201 bouts (1,809 trials).

For the *exploratory* analyses of pupil data, we selected the last five trials before each switch and the first five trials after each switch. The trials around one single switch were treated as belonging to the same *bout* of adjacent trials, with trials nested in bouts and bouts nested in participants. This resulted in a final data set with 247 labor-to-leisure switches (2,225 trials) and 247 leisure-to-labor switches (2,180 trials).

Appendix S2

Participant- and Trial-Based Data Inclusion in Study 2

In line with our pre-registered criteria, we excluded two participants from all our analyses as they only performed the labor task and never switched, and two further participants because their eye-tracking data were unusable due to high rates of missing values. This left a sample of 31 participants that was used in all analyses.

We selected again the last five trials before each switch between tasks and the first five trials after each switch. Here, also the joystick movements in the motor control assessment phase and the observed frame movements in the visual control assessment were treated as switches. In total, we analyzed 272 switches from labor to leisure (2,594 trials), 273 switches from leisure to labor (2,535 trials), and 248 visual switches (2,480 trials).

Regarding the motor control assessment, unexpectedly, participants committed a considerable amount of errors (217 out of 2,480 trials, i.e. 10% of all trials; of which 148 were false-positive switches and 69 false-negative non-switches). As a liberal exclusion criterion, to keep as many correct trials as possible, we decided to first exclude eight participants, as each of their eight bouts, i.e. required movements of the joystick surrounded by ten trials, contained at least one error. This left 39 trials with errors (11 false positives, 28 false negatives), which we deleted in a second step, resulting in 164 remaining bouts (1,880 trials). As a more conservative exclusion criterion, we excluded all bouts with any errors, resulting in 155 bouts (1,550 trials). The pattern of significant and non-significant results was the same for both exclusion criteria. In the main text, we report results based on the liberal exclusion.

Appendix S3

Testing Accuracy Decrements Before Breaks

A potential reason for participants’ decision to switch from the labor to the leisure task might be that they recognize a decrease in their performance, are afraid that they might forfeit their chance for an extra monetary bonus, and thus decide to take a break in order to rest and restore their ability to concentrate. Under this explanation, one might assume that switches could be induced by error monitoring, independent of changes in motivation or NE levels.

However, note that participants did not receive any error feedback during the tasks, and thus had imperfect knowledge about their performance. Furthermore, AGT itself predicts that increased tonic NE levels will lead to decreases in accuracy, which is supported by behavioral evidence: animal work found switches from exploitation to exploration to be accompanied by increased false alarm rates (Cohen, McClure, & Yu, 2007), and increased pupil diameter has been found associated with lapses in attention in humans (Hopstaken, van der Linden, Bakker, & Kompier, 2015; Unsworth & Robison, 2016). Hence, decreases in accuracy might not be an independent mechanism, but a side-effect of changes in NE levels.

In exploratory analyses, we tested whether decreases in accuracy would occur during the last five trials before switches from labor to leisure. We used generalized linear mixed effects models (GLMEMs) in the package lme4 (Bates, Mächler, Bolker, & Walker, 2015) in R (R Core Team, 2017) with accuracy (binary: correct or incorrect) as outcome and trial number relative to switch as predictor. The predictor was standardized. We added a random intercept and a random slope of trial number for each participant and for each bout of each participant to the model, including all possible random correlations. *P*-values were calculated using likelihood ratio tests as implemented in the package afex (Singmann, Bolker, Westfall, & Aust, 2018), since *F*-tests are not available for GLMEMs. We computed 95% confidence intervals via bootstrapping with 1,000 simulations using lme4’s bootMer function, which uses the boot package (version 1.3.18, Canto & Ripley, 2016). Effectively, we analyzed of 376 switches (1,525 trials) in Study 1 and 372 switches (1,591 trials) in Study 2.

In Study 1, there was a non-significant trend of decreasing accuracy during the last five trials before switches from labor to leisure, β = -.10, 95% CI [-.25, .05], χ^2^(1) = 2.85, *p* =.091. In Study 2, there was a significant decrease in accuracy, β = -.26, 95% CI [-.47, -.06], *χ^2^*(1) = 4.90, *p* = .027. We conclude that there was mixed evidence for accuracy decreasing directly before a break.

Appendix S4

Testing Accuracy Restoration After Compared to Before Breaks

As explained in S3, subjects might use breaks as a chance to restore their ability to concentrate. We thus tested in exploratory analyses whether accuracy was significantly better in the first five trials after a break (i.e. a series of leisure trials) compared to the last five trials before a break.

We used GLMEMs with accuracy as outcome and a binary factor indicating whether trials were before or after the leisure bout as the sole predictor. Treatment-coding was applied. We added random intercepts and slopes of trial number for each participant to the model, including all random correlations. Again, *p* values were computed with likelihood ratio tests. We analyzed 247 breaks (2,181 trials) in Study 1 and 272 breaks (2,338 trials) in Study 2.

In Study 1, indeed, mean accuracy was slightly higher during the first five labor trials after a switch from leisure back to labor (*M* = .85, *SD* = .36), than during the last five trials before a switch from labor to leisure (*M* = .80, *SD* = .40), β = .15, 95% CI [.01, .27], χ^2^(1) = 5.23, *p* = .022. In Study 2, however, there was no significant increase in average accuracy from before a break, (*M* = .79, *SD* = .41) to after a break (*M* = .81, *SD* = .39), β = .03, 95% CI [-.11, .16], *χ^2^*(1) = 0.63, *p* = .427. Hence, we obtained mixed evidence for the hypothesis that performing the leisure task had a recreational function and immediately improved participants performance.

Appendix S5

Correlations of Overall Average Baseline Pupil Diameter and Pupil Dilations With Performance Measures Across Both Studies

| Variable | *2* | *3* | *4* | *5* | *6* |
| --- | --- | --- | --- | --- | --- |
| 1. Average pupil baselines | -.37** | -.03* | -.29** | -.12** | -.26** |
| 2. Average pupil dilations | - | -.01* | -.19** | -.01** | -.08** |
| 3. # trials spent on labor |  | - | -.33** | -.35** | -.27** |
| 4. # switches away from labor |  |  | - | -.38** | -.09** |
| 5. % button presses during labor |  |  |  | - | -.38** |
| 6. % correct responses during labor |  |  |  |  | - |
| *Note*. Pearson correlations of aggregated raw baseline pupil diameter and aggregated raw pupil dilations with counts of events (trials, switches) and percentages (button presses, correct responses) during labor trials across both studies (*N* = 62).  * *p* < .05, ** *p* < .01, uncorrected. | | | | | |

Appendix S6

Time Course of Pupil Baseline Diameter and Pupil Dilations in the Motor and Visual Control Assessment in Study 2.

| 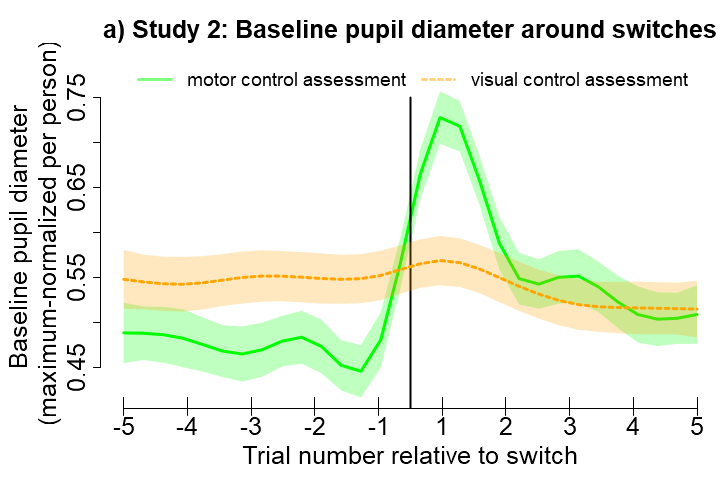 | 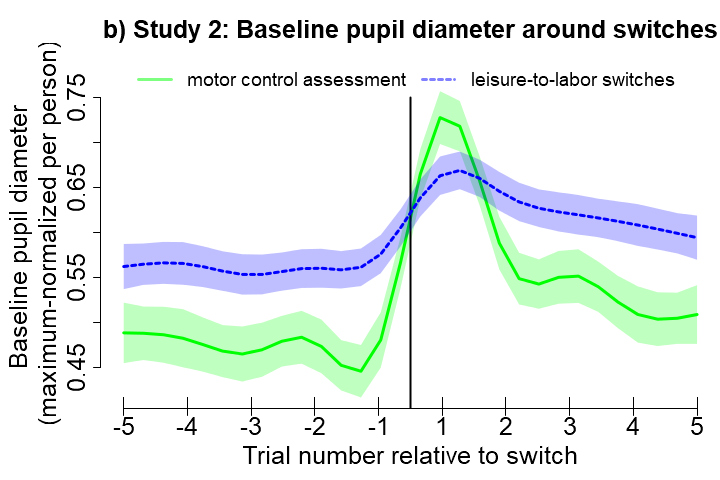 |
| --- | --- |
| 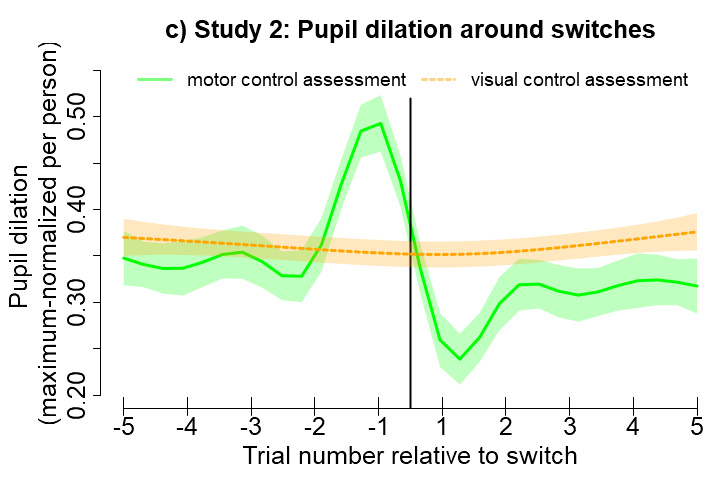 | 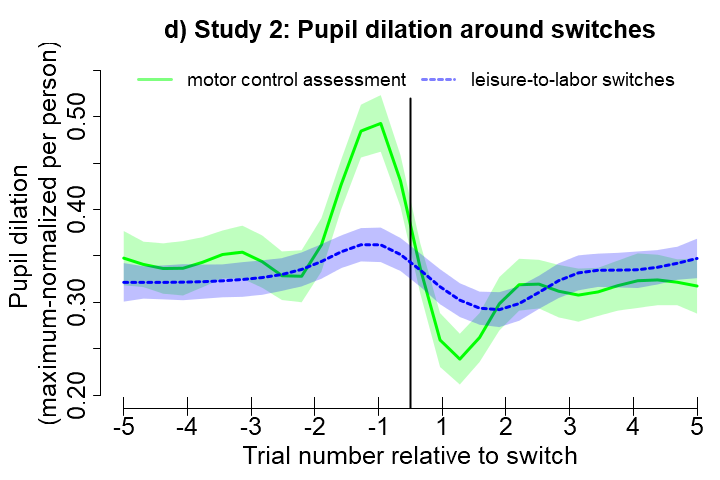 |
| *Figure 3*. Time course of in pupil baseline diameter (a) and pupil dilations (c) in the motor and visual control assessment in Study 2. For both baselines and dilations, there was a significant increase and subsequent decrease around switches in the motor condition, but not in the visual condition. The baseline changes in the motor condition were more peaked, but also more short-lived than those in leisure-to-labor switches (b). The dilation increase in the motor condition was unparalleled in leisure-to-labor switches (d). Plots are based on the GAMMs computed. Shades indicate 95%-CIs. Vertical lines indicate the time point of the switch. | |

Appendix S7

Group-Level Means, Standard Deviations, Cronbach’s Alpha, and Correlations Between Self-Reported Procrastination Tendency and Action- vs. State Orientation

| Measure | *2* | *3* | *4* | *5* | *6* | *M* | *SD* | *α* |
| --- | --- | --- | --- | --- | --- | --- | --- | --- |
| 1. IPS | -.07 | -.67** | -.48** | -.17** | -.76** | 3.01 | 0.62 | .86 |
| 2. ACS-24 AOF | – | .47** | .58** | .17** | .09** | 4.76 | 3.26 | .81 |
| 3. ACS-24 AOD |  | – | .50** | .20** | .59** | 5.98 | 3.28 | .80 |
| 4. SSI-K3 SR |  |  | – | .33** | .28** | 2.57 | 0.42 | .80 |
| 5. SSI-K3 SC |  |  |  | – | .19** | 2.60 | 0.43 | .74 |
| 6. SSI-K3 VD |  |  |  |  | – | 2.52 | 0.48 | .84 |
| *Note*. Higher IPS values indicate higher procrastination tendencies, and higher values on the other scales indicate higher action orientation. Samples of both studies (total *N* = 62) were combined. IPS = Irrational Procrastination Scale; ACS-24 AOF = ACS-24 Action orientation subsequent to failure vs. preoccupation subscale; ACS-24 AOD = ACS-24 Prospective and decision-related action orientation vs. hesitation subscale; SSI-K3 SR = SSI-K3 Self-regulation (Competence) subscale; SSI-K3 SC = SSI-K3 Self-control subscale; SSI-K3 VD = SSI-K3 Volitional development (Action development) subscale. | | | | | | | | |

* *p* < .05, ** *p* < .01, uncorrected.

**References**

Bates, D., Mächler, M., Bolker, B., & Walker, S. (2015). Fitting linear mixed-effects models using lme4. *Journal of Statistical Software*, *67*(1), 1–48. doi: 10.18637/jss.v067.i01

Canto, A., & Ripley, B. (2016). boot: Bootstrap R (S-Plus) functions.

Cohen, J. D., McClure, S. M., & Yu, A. J. (2007). Should I stay or should I go? How the human brain manages the trade-off between exploitation and exploration. *Philosophical Transactions of the Royal Society of London. Series B, Biological Sciences*, *362*(1481), 933–942. doi: 10.1098/rstb.2007.2098

Hopstaken, J. F., van der Linden, D., Bakker, A. B., & Kompier, M. A. J. (2015). The window of my eyes: Task disengagement and mental fatigue covary with pupil dynamics. *Biological Psychology*, *110*, 100–106. doi: 10.1016/j.biopsycho.2015.06.013

R Core Team. (2017). *R: A language and environment for statistical computing*. *R Foundation for Statistical Computing*. Vienna, Austria: R Foundation for Statistical Computing. Retrieved from www.R-project.org

Singmann, H., Bolker, B., Westfall, J., & Aust, F. (2018). afex: Analysis of factorial experiments. Retrieved from https://cran.r-project.org/package=afex

Unsworth, N., & Robison, M. K. (2016). Pupillary correlates of lapses of sustained attention. *Cognitive, Affective, & Behavioral Neuroscience*, *16*(4), 601–615. doi: 10.3758/s13415-016-0417-4
